# Supplementary material for: Helicobacter pylori Exploit Short-Chain Fatty Acids-Induced CAPZA1 Overexpression to Emerge CD44v9-Positive Stemness
Source: Gastro Hep Adv. 2025 Dec 10;5(3):100860. doi: 10.1016/j.gastha.2025.100860 (PMC12828395; doi:10.1016/j.gastha.2025.100860)
Supplement: Figure A1 and Table A1 [file mmc1.pdf]

Supplementary information

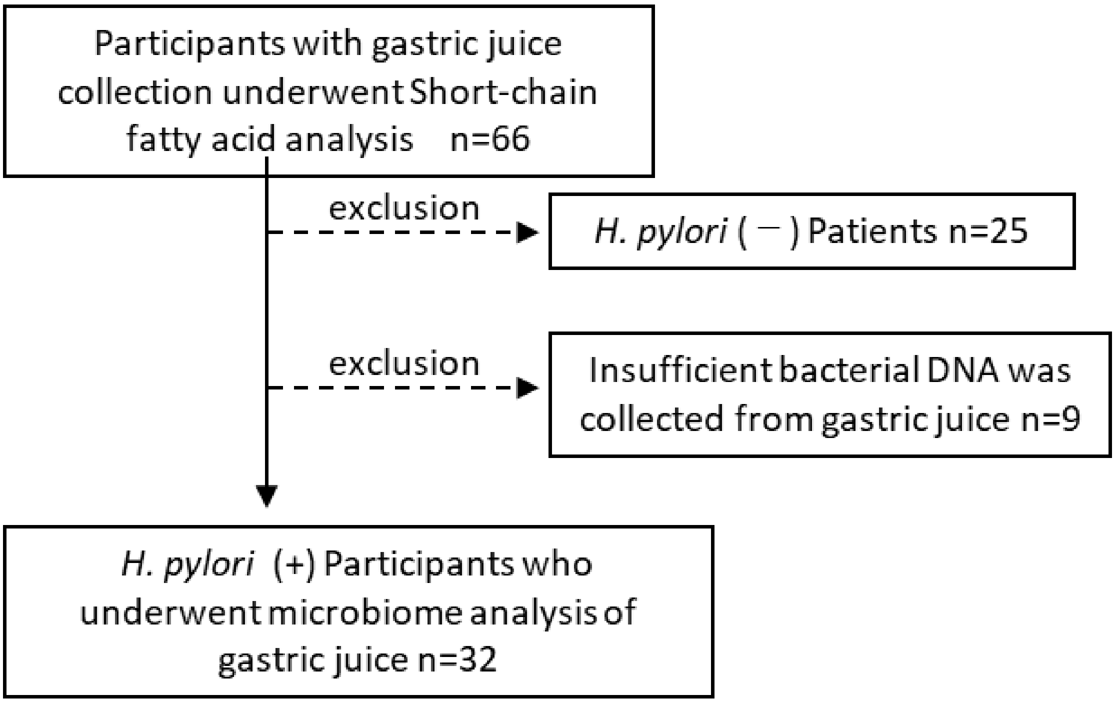

Supplementary Figure S1.

The inclusion criteria and study flow diagram. A total of 66 participants (44 male and 22 female) were enrolled. Of all participants, gastric juice microbiome analysis was performed on 32 individuals, excluding 25 uninfected with *H. pylori* and 9 individuals for whom insufficient bacterial DNA in the gastric juice could not be collected.

Supplementary Table S1.

Table S1. Basic patient characteristics by presence or absence of gastric cancer

	H. pylori + GC (+) (n=11)	*H. pylori* + GC (-) (n=30)	*P* value
Age (mean years± SD)	68.3±17.4	73.2±10.9	N.S. (.81)
BMI (mean years± SD)	20.2 ± 2.8	23.4 ± 2.8	< .01
Men n, (%)	8 (72.7)	22 (73.3)	N.S. (> .99)
Female n, (%)	3 (27.3)	8 (26.7)	
H. pylori current infection n, (%)	6 (54.5)	5 (16.7)	
H. pylori past infection n, (%)	5 (45.5)	25 (83.3)	
PPI/P-CAB n, (%)	5 (45.5)	11 (36.7)	N.S. (> .72)
