# Supplementary material for: Helicobacter pylori Exploit Short-Chain Fatty Acids-Induced CAPZA1 Overexpression to Emerge CD44v9-Positive Stemness
Source: Gastro Hep Adv. 2025 Dec 10;5(3):100860. doi: 10.1016/j.gastha.2025.100860 (PMC12828395; doi:10.1016/j.gastha.2025.100860)
Supplement: Extended PDF [file mmc2.pdf]

Gastro Hep Advances 2026;5:100860

ORIGINAL RESEARCH—BASIC

Helicobacter pylori Exploit Short-Chain Fatty Acids-Induced CAPZA1 Overexpression to Emerge CD44v9-Positive Stemness

Hitoshi Tsugawa,[1,2,*] Jin Imai,[2,3,*] Eiji Sugiyama,[4,5] Chanudporn Sugiyama,[4,6] Takashi Ueda,[7] Miwa Hirai,[7] Kenichiro Todoroki,[4] and Hidekazu Suzuki[7]

[1]Transkingdom Signaling Research Unit, Division of Host Defense Mechanism, Tokai University School of Medicine, Isehara, Kanagawa, Japan; [2]Institute of Medical Sciences, Tokai University, Isehara, Kanagawa, Japan; [3]Department of Clinical Health Science, Tokai University School of Medicine, Isehara, Japan; [4]School of Pharmaceutical Sciences, University of Shizuoka, Suruga-ku, Shizuoka, Japan; [5]Laboratory of Analytical Chemistry, Faculty of Pharmacy, Meijo University, Tempaku, Nagoya, Japan; [6]Graduate School of Bioagricultural Sciences, Nagoya University, Chikusa, Nagoya, Japan; and [7]Division of Gastroenterology, Department of Internal Medicine, Tokai University School of Medicine, Isehara, Japan.

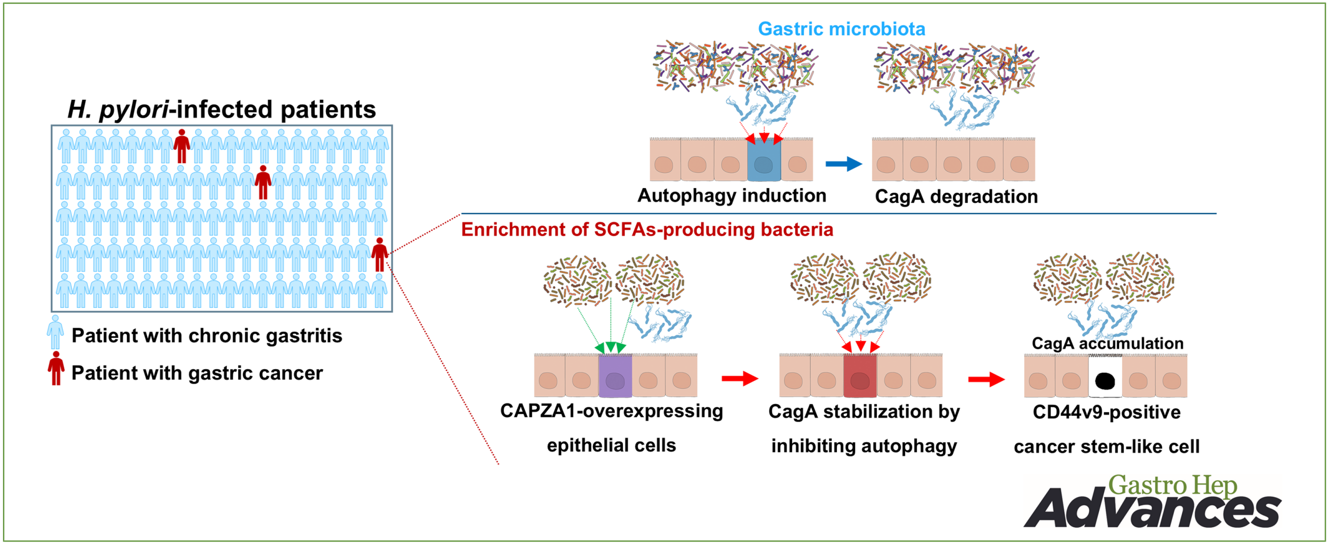

BACKGROUND AND AIMS: Although *Helicobacter pylori* infects a large proportion of the global population, only a small subset of the infected individuals develop gastric cancer. The molecular mechanisms underlying the selective progression of gastric carcinogenesis are not fully understood. This study aimed to elucidate these mechanisms by focusing on CD44v9-positive cell generation in *H pylori*–infected gastric mucosa. **METHODS:** Using *H pylori* infection models in human gastric adenocarcinoma cells, mice, and mouse-derived gastric organoids, we examined the effects of short-chain fatty acids (SCFAs) on the induction of CD44v9-positive cells using western blotting and immunofluorescence. SCFA concentrations and microbiota compositions were analyzed in gastric juice samples from *H pylori*–infected patients to evaluate their association with gastric cancer risk. **RESULTS:** Propionate and butyrate induced capping actin protein of muscle Z-line α subunit 1 (CAPZA1) overexpression via histone deacetylase inhibition. In SCFA-induced CAPZA1-overexpressing cells, the *H pylori*–derived oncoprotein CagA accumulated due to impaired autophagic degradation, leading to enhanced CD44v9 expression. In the gastric antrum, CD44v9-positive cells undergo CagA-mediated stem cell transformation, whereas a distinct signaling mechanism operates in the gastric corpus. In patients with early gastric cancer, intragastric concentrations of propionate and butyrate are elevated, and the microbiota is enriched with SCFA-producing bacteria. **CONCLUSION:** SCFA-induced CAPZA1-overexpressing cells serve as a scaffold niche that supports CagA activity and promotes CD44v9-positive cancer stem-like cells. This study sheds new light on the early molecular events driving *H pylori*–associated gastric carcinogenesis and may inform future strategies for early detection and intervention.

*Contributed equally to this work.

Abbreviations: But, butyrate; CAPZA1, capping actin protein of muscle Z-line α subunit 1; *H pylori*, *Helicobacter pylori*; HDAC, histone deacetylase; Pro, propionate; SCFAs, short-chain fatty acids; SPF, specific pathogen-free.

Most current article

Copyright © 2025 The Authors. Published by Elsevier Inc. on behalf of the AGA Institute. This is an open access article under the CC BY license (http://creativecommons.org/licenses/by/4.0/).
2772-5723
https://doi.org/10.1016/j.gastha.2025.100860

Keywords: Autophagy; CagA; Cancer-Stem Cells; Gastric Cancer

Introduction

Although *Helicobacter pylori* infection is a major risk factor for gastric cancer (GC), only 1%–3% of infected individuals ultimately develop GC,[1] indicating that GC arises only in a subset of infected individuals and implies the involvement of additional host or environmental factors other than *H pylori*. However, the molecular mechanisms underlying the selection of individuals with GC from *H pylori*–infected patients remain to be fully elucidated. A study using an insulin-gastrin transgenic mouse model of GC demonstrated that *H pylori* infection under specific pathogen-free (SPF) conditions leads to GC, whereas infection under germ-free conditions does not induce tumorigenesis.[2] These findings indicate that the presence of specific gastric commensal bacteria is essential for *H pylori*–driven carcinogenesis. Many analyses of the human gastric microbiota have been performed using gastric juice samples; however, the specific bacterial taxa that contribute to gastric carcinogenesis in the presence of *H pylori* have not been clearly identified.

We previously demonstrated that the generation of CD44v9-positive cells in *H pylori*–infected gastric mucosa significantly increased the risk of metachronous recurrence following endoscopic resection of early GC.[3] One underlying mechanism involves the *H pylori*–derived oncoprotein CagA, which evades autophagic degradation in CD44v9-positive gastric epithelial cells, resulting in intracellular stabilization.[4] Our previous findings indicated that the emergence of CD44v9-positive cells in *H pylori*–infected gastric mucosa represents a critical host-related factor that promotes gastric carcinogenesis. We also demonstrated that CD44v9-positive cells originate from capping actin protein of muscle Z-line α subunit 1 (CAPZA1)-overexpressing gastric epithelial cells following the accumulation of CagA, which occurs due to autophagy inhibition that impairs CagA degradation in these cells.[5] CAPZA1 expression is epigenetically regulated by histone acetylation and is significantly upregulated by histone deacetylase (HDAC) inhibitors.[6]

Short-chain fatty acids (SCFAs), including acetate, propionate (Pro), and butyrate (But), are microbial metabolites produced during dietary fiber fermentation by intestinal bacteria. These SCFAs are present in high concentrations in the gastrointestinal tract and act as endogenous HDAC inhibitors.[7,8] In addition to their local effects, SCFAs enter the systemic circulation and influence a wide range of host physiological functions, including cancer development, immune regulation, metabolic homeostasis, and cardiovascular and nervous system health maintenance.[8]

Based on these findings, we hypothesized that SCFAs induce CAPZA1-overexpressing cell production in *H pylori*–infected gastric mucosa, leading to CD44v9-positive cancer stem-like cell development through stabilization of CagA in CAPZA1-overexpressing cells. In this study, we tested this hypothesis using a multidisciplinary research approach, including *in vitro* analysis using human gastric adenocarcinoma (AGS) cells, *ex vivo* studies using gastric organoids, *in vivo* experiments using a mouse infection model, and validation using human clinical specimens. Our study findings show that SCFAs provide a foothold niche for epithelial cells for CagA stabilization and activity by generating CAPZA1-overexpressing cells and facilitating gastric epithelial cell transformation into CD44v9-positive cancer stem-like cells. These results elucidate the early molecular events driving *H pylori*–associated gastric carcinogenesis and may inform future strategies for early detection and intervention.

Materials and Methods

Ethics Statement

Animal care and use followed the regulations of "Act on Welfare and Management of Animals of Japan," "Standards relating to the Care and Keeping and Reducing Pain of Laboratory Animals," "Standards relating to the Methods of Destruction of Animals," "Guidelines for Proper Conduct of Animal Experiments," and "Fundamental Guidelines for Proper Conduct of Animal Experiments." All animal experiments were approved by the Keio University (Tokyo, Japan) Animal Research Committee (no. 19048) and the Tokai University (Kanagawa, Japan) Animal Research Committee (no. 250086 and 250,118). The study protocols for human gastric juice sample analysis were approved by the Research Ethics Committee of the Tokai University School of Medicine (20R-365). The study was conducted in accordance with the principles of the Declaration of Helsinki. All authors had access to the study data and had reviewed and approved the final manuscript.

Drugs and Antibodies

LysoTracker Red DND-99 (Thermo Fisher Scientific, Waltham, MA, USA, cat# L7528) was used to stain the lysosomes. Pro acid (cat# 163–04,726) and But acid (cat# 023–05,396) were purchased from Wako Pure Chemical Industries, Ltd. (Tokyo, Japan), adjusted to pH 7.0, and administered to AGS cells, murine stomach-derived organoids, and mice. The following antibodies were used for western blotting: anti-CAPZA1 (Merck Millipore, Billerica, MA, cat# AB6016; 1:2000), anti-CagA (Austral Biologicals, San Ramon, CA, cat# HPM-5001–5; 1:2000), anti-LAMP1 (Santa Cruz Biotechnology, Santa Cruz, CA, cat# sc-20,011; 1:2000), anti-CD44 (Sigma-Aldrich, St. Louis, MO, cat# HPA005785; 1:1000), anti-GAPDH (Cell Signaling Technologies, Danvers, MA, cat# 2118S; 1:2000), and anti-β-actin (Sigma-Aldrich, cat# A5316; 1:2000). The following antibodies were used for fluorescence immunocytochemistry: anti-CAPZA1 (OTI2G4) (Thermo Fisher Scientific, cat# MA5-25093; 1:500 or Abcam, Cambridge, UK, cat# ab166892; 1:500), anti-CD44v9 (Cosmo Bio, Tokyo, Japan, cat# CAC-LKG-M001; 1:500), anti-CagA (Austral Biologicals, cat# HPM-5001–5; 1:500), anti-LGR5 (Atlas antibodies, Stockholm, Sweden, cat# HPA012530; 1:200), anti-KLF5 (GeneTex, Irvine, CA, cat#GTX103289; 1:200), anti-SALL4 (Abcam, cat# ab29112; 1:200), and anti-trefoil factor 2 (TFF2) (Proteintech, Wuhan, China, cat# 13681-1-AP; 1:200). 4',6-diamidino-2-phenylindole was used to visualize nuclei.

Bacterial Culture

H pylori strains ATCC700392, TN2GF4, G27, and the G27 *cag*PAI-deleted isogenic mutant (Δ*cag*PAI) were cultured on Columbia HP (Becton-Dickinson, Sparks, MD, cat#251268) or sheep blood (Becton-Dickinson, cat# 251148) agar for 2 days at 37 °C under microaerobic conditions, which were maintained using AnaeroPack MicroAero (Mitsubishi Gas, Tokyo, Japan; cat# A-28). TN2GF4 strains were kindly gifted from Dr Hidenori Matsui, and G27 and G27 Δ*cagPAI* stains were gifted from Dr Masanori Hatakeyama and Dr Naoko Murata-Kamiya.[9] The bacterial strains were stored at −80 °C in Brucella Broth (Becton-Dickinson) containing 25% glycerol.

Cell Culture

The human GC cell line, AGS, was purchased from the American Type Culture Collection (Rockville, MA) and cultured in RPMI 1640 medium (Invitrogen, Carlsbad, CA; cat#11875093) supplemented with 10% fetal bovine serum.

Murin Organoid Culture and Mucosoid Construction

The following drugs were used for gastric organoid construction and culture; liberase TH (Sigma-Aldrich, cat# 5401151001), Glutamax (Thermo Fisher Scientific, cat# 35050061), HEPES (Thermo Fisher Scientific, cat# 15630080), Afamin/Wnt3a CM (MBL life Science, Tokyo, Japan, cat# J-ORMW301R), recombinant mouse EGF (Thermo Fisher Scientific, cat# PMG8043), recombinant human FGF10 (PeproTech, Rocky Hill, NJ, cat# 100-26), recombinant human R-Spondin1 (R&D Systems, Minneapolis, MN, cat# 4645-RS-100), recombinant mouse Noggin (PeproTech, cat# 250-38), human gastrin 1 (Sigma-Aldrich, cat# G9145-0.5MG), N-Acetyl-L-cysteine (Sigma-Aldrich, cat# A9165-5G), B-27 supplement (Thermo Fisher Scientific, cat# 17504044), A83-01 (ALK inhibitor) (R&D Systems, cat# 2939), Y-27632 (ROCK inhibitor) (Nacalai Tesque, Kyoto, Japan, cat# 18190-96), Corning Matrigel Matrix (Corning, Corning, NY, cat# 354230), advanced DMEM/F12 (Thermo Fisher Scientific, cat# 12634010), and Corning Cell Recovery Solution (Corning, cat# 354253). We constructed polarized epithelial monolayers of the gastric mucosa, called mucosoids, from murine organoids derived from the murine gastric corpus and antrum according to a previously reported method.[10] Briefly, mouse gastric tissues were washed in phosphate-buffered saline (PBS), trimmed to remove fat and connective tissue, and cut into approximately 1 mm^2 fragments. Tissue fragments were enzymatically digested in 3–5 mL of 50 μg/mL Liberase TH at 37 °C for 30 minutes. The supernatant containing the dissociated cells was collected and centrifuged at 200× g for 3 minutes. The resulting pellet was washed, resuspended in cold Matrigel, and seeded onto prewarmed culture plates. The plates were incubated at 37 °C for 5–10 minutes to allow the Matrigel to solidify. Advanced DMEM/F12 medium containing each supplement, as described earlier, was added to the plate wells to initiate the culture. The medium was refreshed every 2–3 d. For the construction of mucosoids, Matrigel domes were dissolved in a cell-recovery solution (Corning, cat# 354253). The suspension was centrifuged at 300× g for 3 minutes, and the pellet was trypsinized. The cell pellets were resuspended in advanced DMEM/F12 medium containing each supplement and seeded onto collagen-coated Transwell inserts (Merck-Millipore, Billerica, MA, cat# PIHP01250) in 24-well culture plates.

In Vitro H pylori *Infection*

AGS cells were infected with *H pylori* strains ATCC 700392, G27 (*H pylori* G27), or a cag pathogenicity island (*cag*PAI)-deleted isogenic mutant (*H pylori* G27 Δ*cag*PAI) at a multiplicity of infection of 50 for 5 h. Following infection, the cells were washed with PBS and subsequently incubated in RPMI 1640 medium containing 400 μg/mL kanamycin for 24 h to kill the bacteria and prevent further injection of CagA.

In Vivo H pylori *Infection*

To eliminate the influence of endogenous SCFAs derived from the gut microbiota, 6- to 8-week-old mice were administered a broad-spectrum antibiotic cocktail in their drinking water for 4 weeks prior to *H pylori* infection, as previously described.[11,12] The antibiotic cocktail consisted of ampicillin (1 g/L; Sigma-Aldrich, cat# A0166), metronidazole (1 g/L; Sigma-Aldrich, cat# M1547), neomycin (1 g/L; Sigma-Aldrich, cat# N1876), and vancomycin (0.5 g/L; Wako, cat# 222–01,303). Mice were subsequently administered drinking water supplemented with 300-mM SCFAs (either sodium Pro or sodium But) for 1 week, following a previously published protocol.[12] Mice were then orally inoculated with *H pylori* strain TN2GF4 (1×10^8 bacteria). Twenty-eight days post-infection, the mice were euthanized, and their stomachs were harvested. To confirm *H pylori* colonization, viable bacteria were quantified by plating the homogenized stomach tissue on Nissui *Helicobacter* agar (Nissui Pharmaceutical, Tokyo, Japan) and determining the number of colony-forming units.

For immunohistochemistry, tissue sections (4 μm thick) were fixed in 4% paraformaldehyde, deparaffinized, and rehydrated using a graded ethanol series. Antigen retrieval was performed by heating the sections at 105 °C for 10 minutes in the Target Retrieval Solution (pH 9.0; Dako). The sections were then incubated overnight at 4 °C with the appropriate primary antibodies. Immunoreactivity was visualized using Alexa Fluor-conjugated secondary antibodies. Fluorescence signals were analyzed using a Zeiss LSM700, LSM710, or LSM880 confocal microscope.

Organoid H pylori *Infection*

H pylori G27 or G27 Δ*cag*PAI strains were added to mucosoid cultures grown as two-dimensional (2D) monolayers on Transwell inserts at a multiplicity of infection of 100 and incubated for 2 h. After incubation, nonadherent bacteria were removed by washing with PBS, and the medium was replaced with Advanced DMEM/F12. The cells were subsequently cultured for 3 days, and the medium was refreshed daily. Mucosoids were fixed in 4% paraformaldehyde overnight at 4 °C and embedded in paraffin. Mucosoid sections (4 μm) were deparaffinized, rehydrated using a graded ethanol series, and stained with hematoxylin and eosin (H&E). For immunohistochemistry, antigen retrieval was performed by heating the sections at 105 °C for 10 minutes in Target Retrieval Solution (pH 9.0; Dako, Carpinteria, CA; cat# S2375). Sections were

then incubated with primary antibodies overnight at 4 °C, followed by incubation with Alexa Fluor-conjugated secondary antibodies for 1 h at room temperature. Fluorescent images were acquired using an LSM710, LSM700, or LSM880 confocal microscope (Carl Zeiss, Oberkochen, Germany). Staining intensity was quantified using the ImageJ software (National Institutes of Health, Bethesda, MD, USA).

HDAC Activity

Nuclear protein fractions were extracted using a subcellular protein fractionation kit (Thermo Fisher Scientific, cat# 78840), according to the manufacturer's instructions. HDAC activity was measured using an HDAC Activity Assay kit (BioVision, Milpitas, CA, cat# K331).

Chromatin Immunoprecipitation Assay

Immunoprecipitation was performed using a Simple Chromatin Immunoprecipitation (ChIP) Plus Sonication Chromatin IP Kit (cat #56383S; Cell Signaling Technology) according to the manufacturer's protocol. Chromatin crosslinking was performed using 1% formaldehyde in PBS. The input fractions were collected before immunoprecipitation and used as positive controls. Supernatants were incubated overnight at 4 °C with anti-histone H3 (acetyl K9) antibody (Abcam, cat# ab4441) or control rabbit IgG (Sigma-Aldrich, cat# 18140). The enriched genomic DNA was subsequently analyzed by quantitative PCR using the EpiTect ChIP quantitative PCR Primer Assay for *CAPZA1* (Qiagen, Valencia, CA, cat# GPH100073(−)01A), which targets a region approximately 1 kb upstream of the transcription start site. The primers used in this assay were designed and optimized to detect DNA enrichment in the specified promoter region.

Western Blot

Total protein (10 μg per lane) was resolved by electrophoresis on a 10% Bis-Tris Plus Gel (Invitrogen, Carlsbad, CA, Cat# NW00107BOX). β-actin or GAPDH was used as a loading control, detected using specific primary antibodies. Immunoreactive bands were visualized by enhanced chemiluminescence using ECL Prime western blotting detection reagents (Cytiva, Uppsala, Sweden, Cat# RPN2232). Band intensities were quantified using the ImageJ software (National Institutes of Health).

Human Participants

Sixty-six participants (44 male and 22 female) scheduled to undergo endoscopy were enrolled in this study at Tokai University Hospital in Kanagawa, Japan. Eligible subjects aged 33–90 years were enrolled. Age, sex, medical history, history of alcohol consumption and smoking, endoscopic features (atrophy, metaplasia, early GC, advanced GC, gastric polyps, superficial gastritis, reflux esophagitis, gastric ulcer scars, and duodenal ulcer scars), and histopathology information were collected. Kyoto classification[13] and pathological findings were used to evaluate gastritis. Early GC is defined as adenocarcinoma of the stomach confined to the mucosa or submucosa, regardless of the presence or absence of lymph node metastasis.[14] Human gastric acid samples were collected from the patients who provided informed consent.

Gastric Microbiota Analysis

Approximately 10 mL of human gastric juice was collected during the upper endoscopy. Human gastric juice samples were centrifuged at 14,000×g for 10 minutes at 4 °C. Total DNA was extracted from the resulting pellets using Nucleospine DNA stool (TaKaRa, Tokyo, Japan, cat# U0472). The V3–V4 region of the 16S rRNA gene was amplified using primers 341F (5′-CCTACGGGNGGCWGCAG-3′) and 805R (5′-GACTACHVGGGTATCTAATCC-3′), synthesized based on the design by Klindworth *et al.*[15] PCR amplification was performed using KAPA HiFi HotStart ReadyMix (Roche; Cat# 07958927001) following the protocol described in the 16S Metagenomic Sequencing Library Preparation guide (Illumina, Document #15044223 B). The resulting libraries were sequenced on an Illumina MiSeq platform using a MiSeq Reagent Kit v3 (Illumina; Cat# MS-102-3003). The reads obtained from sequencing were processed using QIIME2 (version 2022.2).[16] Adapter sequences were trimmed using Cutadapt with an error rate of 0.1, while other parameters were set to default. Denoising, dereplication, and chimera removal were performed using the DADA2 plugin, resulting in amplicon sequence variants. Taxonomic classification was conducted using a naïve Bayes classifier trained on the SILVA 138 database (99% operational taxonomic units) and extracted to match the V3–V4 region of the 16S rRNA gene.[17–19] A phylogenetic tree was constructed based on multiple sequence alignment and approximate maximum likelihood methods, using a sampling depth of 12,000.[20,21] Alpha rarefaction analysis confirmed that the sequencing depth was sufficient to capture microbial diversity. Principal coordinate analysis (PCoA) was performed using the distance matrix derived from the phylogenetic tree. Visualization and downstream statistical analyses were conducted in R (version 4.3.0) using the qiime2R, phyloseq, and ggplot2 packages. Taxonomic abundance data collapsed to the family level were used for differential abundance testing using linear discriminant analysis effect size method and differential expression sequencing 2.[22,23]

SCFA Content Determination in Human Gastric Juice

A certified reference solution containing SCFAs (CRM46975, Merck, Darmstadt, Germany) was used for preparing standard solutions. Internal standard solution containing acetic-2,2,2-d_3 acid (C/D/N isotope, Pointe-Claire, Canada), propionic-3,3,3-d_3 acid (C/D/N isotope), butyric-4,4,4-d_3 acid (C/D/N isotope), pentanoic-5,5,5-d_3 acid (C/D/N isotope), hexanoic-6,6,6-d_3 acid (C/D/N isotope), and dl-lactate-d_3 (Toronto Research Chemicals, Vaughan, Canada) was used for checking chromatographic separation and for normalizing peak area.

To prepare a standard solution for calibration curve, the reference solution of SCFAs was diluted, and a 3-fold serial dilution was performed to prepare solutions at 2.2–60 μM. After transferring 200 μL of each solution to a new tube, 20 μL of 100 μM internal standard solution and 980 μL of acetonitrile (MeCN) were added. The prepared solutions containing

SCFAs (0.37–10 μM) and internal standards (1.7 μM) were used for the following derivatization procedure.

The supernatant of human gastric juice centrifuged as described above was filtered by a 0.45-μm membrane filter and was stored at −80 °C until use. After the frozen supernatant was thawed on ice, 120 μL of each was diluted with 120 μL of H_2O. Two-hundred μL of the 2-fold diluted supernatant and that of the undiluted supernatant were independently transferred to new plastic tubes, mixed with 100-μM internal standard solution (20 μL) and MeCN (980 μL), sonicated for 1 minute, then centrifuged at 10,000×g for 5 minutes at 4 °C. Forty μL of the supernatants were transferred to new tubes for derivatization of SCFAs.

The derivatization was performed as follows: 40 μL of either standard solution or the gastric juice samples was mixed with 20 μL of 120 mM 1-Ethyl-3-(3-dimethylaminopropyl)carbodiimide containing 6% pyridine in 50% acetonitrile and 20 μL of 200-mM 3-nitrophenylhydrazine hydrochloride in 50% MeCN. The mixture was vortexed, incubated at 40 °C for 30 minutes, filtered through a 0.45-μm membrane filter.

The derivatized SCFAs were analyzed using an UPLC system (ACQUITY UPLC I-Class, Waters, Milford, MA, USA) equipped with a triple quadrupole MS (Xevo TQ-S, Waters). An ACQUITY UPLC BEH C18 column (1.7 μm, 100 mm × 2.1 mm i.d., Waters) connected with a guard column (ACQUITY UPLC BEH C18 VanGuard Pre-column, 1.7 μm, 2.1 mm × 5 mm, Waters) was used for the separation. The SCFA derivatives were analyzed using water containing 0.01% formic acid (v/v) and MeCN containing 0.01% formic acid (v/v) as mobile phases A and B, respectively. The samples were maintained at 10 °C in the autosampler, and the injection volume was set at 1 μL. The LC separation was performed under a gradient elution as follows: 15%–15% B (0.0–2.0 minutes), 15%–20% B (2.0–10.0 minutes), 20%–60% B (10.0–15.0 minutes), 60%–100% B (15.0–16.0 minutes), 100%–100% B (16.0–17.0 minutes), 100%–15% B (17.0–17.1 minutes), 15%–15% B (17.1–20.0 minutes). The flow rate was set at 0.350 mL/min, and the column temperature was maintained at 40 °C. The conditions for MS/MS were set as follows: ion mode, negative; capillary voltage, 2.5 kV; desolvation gas flow, 1000 L/h; cone gas flow, 150 L/h; nebulizer gas flow, 7.0 bar; source temperature, 150 °C; desolvation temperature, 500 °C; and data acquisition mode, multiple reaction monitoring (MRM). The MRM parameters were set as follows: cone voltage, 40 V (for all analytes); MRM transition, m/z 208.1→137.1 (propionic acid derivative), m/z 222.1→137.1 (butyric acid derivative and isobutyric acid derivative), m/z 211.1→137.1 (propionic acid-d_3 derivative), m/z 225.1→137.1 (butyric acid-d_3 derivative); collision energy, 20 eV (for all analytes). When both the SCFA concentrations in the undiluted and 2-fold diluted gastric juice samples were quantifiable, the mean of the two values was used as the final determined concentration. Data were analyzed using MassLynx V4.2 (Waters).

Statistical Analysis

Data are presented as mean ± standard deviation (SD). Means of multiple groups were compared using analysis of variance (ANOVA), followed by Tukey's tests using JSTAT statistical software (version 8.2) or SPSS version 22 for Windows (SPSS, Chicago, IL, USA). $P < .05$ was considered significant.

Results

SCFAs Induce CAPZA1 Expression in Gastric Epithelial Cells by Inhibiting HDAC Activity, Developing CD44v9 Expression under H pylori Infection

We investigated the effects of SCFAs on CAPZA1 expression in gastric epithelial cells. Treatment with Pro or But to AGS cells significantly increased the CAPZA1 expression in a dose-dependent manner (Figure 1A). Given the established role of SCFAs as HDAC inhibitors[24,25] and our previous finding that *CAPZA1* mRNA expression is regulated by the acetylation of its promoter region,[26] we examined whether Pro or But modulates *CAPZA1* promoter region acetylation using ChIP with an antibody against acetylated histone H3 in AGS cells. HDAC activity in AGS cells was significantly decreased by treatment with both Pro and But (Figure 1B), and acetylated histone H3 levels increased upon Pro or But treatment (Figure 1C). Enhanced CAPZA1 expression suppresses CagA-mediated autophagy by inhibiting lysosome.[6] We examined whether CagA-degrading autophagy was inhibited. Enhanced LysoTracker Red staining, a marker of lysosomes in AGS cells after *H pylori* infection, was repressed by But treatment (Figure 1D). Furthermore, the intracellular levels of CagA in *H pylori*–infected AGS cells increased in a But concentration-dependent manner (Figure 1E). We previously demonstrated that lysosomal-associated membrane protein 1 (LAMP1) upregulation is essential for autolysosome formation and subsequent CagA degradation, and that CAPZA1 overexpression suppresses LAMP1 expression.[6] Notably, treatment with But at concentrations ≥5 mM resulted in LAMP1 downregulation and a concomitant increase in CAPZA1 expression (Figure 1E). Our previous reports showed that translocated CagA accumulation in CAPZA1-overexpressing cells drives CD44v9 expression.[5] Thus, we examined whether Pro or But treatment induced CD44v9 expression in *H pylori*–infected AGS cells. Treatment with But or Pro induced CD44s and CD44v expression in *H pylori*–infected AGS cells, but not in those infected with a CagA-deletion mutant strain (*H pylori* G27 ⊿ *cag*PAI) (Figure 1F). Collectively, our findings show that in AGS cells, SCFAs induce the overexpression of CAPZA1 by accelerating the acetylation of histones in its promoter region and promoting CD44v9 expression by accumulating CagA under *H pylori* infection.

SCFAs Promote CD44v9-Positive Cell Development in H pylori–Infected Murine Gastric Mucosa

To explore the contribution of SCFAs to the CD44v9-positive cell development in *H pylori*–infected mice, we used an *H pylori*–infected mouse model in which endogenous SCFAs were depleted by antibiotic treatment.[27] SPF mice were administered antibiotics (1 g/L ampicillin, 1 g/L

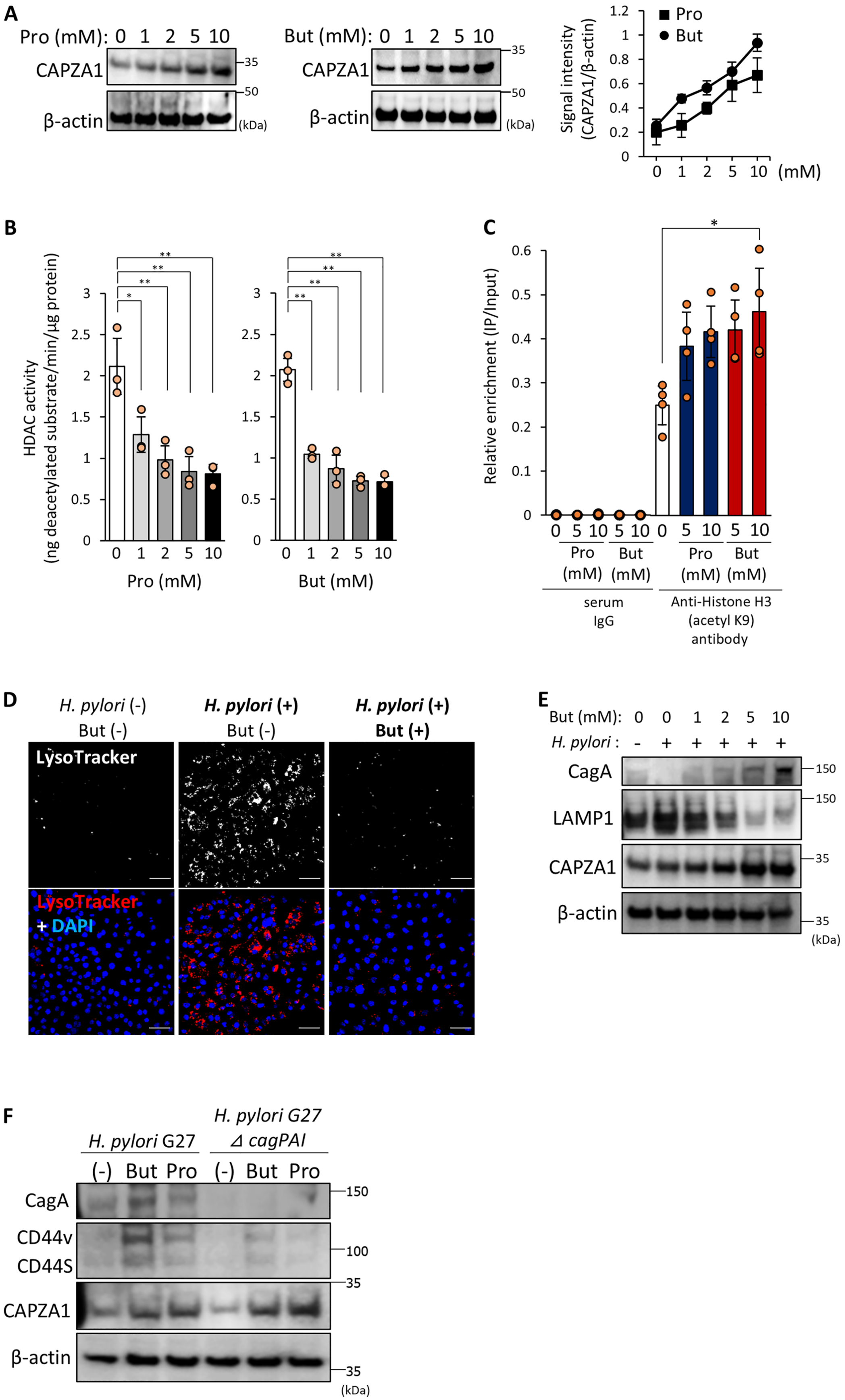
A
Pro (mM): 0 1 2 5 10
CAPZA1
β-actin
But (mM): 0 1 2 5 10
CAPZA1
β-actin
(kDa)
Signal intensity (CAPZA1/β-actin)
Pro
But
(mM)
B
HDAC activity (ng deacetylated substrate/min/µg protein)
Pro (mM)
But (mM)
C
Relative enrichment (IP/Input)
serum IgG
Anti-Histone H3 (acetyl K9) antibody
D
H. pylori (-) But (-)
H. pylori (+) But (-)
H. pylori (+) But (+)
LysoTracker
LysoTracker + DAPI
E
But (mM): 0 0 1 2 5 10
H. pylori : - + + + + +
CagA
LAMP1
CAPZA1
β-actin
F
H. pylori G27
H. pylori G27 ⊿ cagPAI
(-) But Pro (-) But Pro
CagA
CD44v
CD44S
CAPZA1
β-actin

metronidazole, 1 g/L neomycin, and 0.5 g/L vancomycin) in drinking water for 4 weeks, followed by oral supplementation with 300 mM Pro or But since 1 week prior to *H pylori* infection (Figure 2A). Antibiotic treatment significantly suppressed CAPZA1 immunostaining in both the gastric corpus and antrum, whereas Pro or But administration elevated CAPZA1 staining levels in both regions (Figure 2B and C). Furthermore, strong CAPZA1 staining in Pro- or But-administered mice was observed throughout the gastric epithelial mucosa from the base to the pit (Figure 2B). We then examined the alteration of HDAC activity and the levels of acetylated histones in the CAPZA1 proximal promoter region in the gastric epithelial mucosa of But-administered mice. While antibiotic treatment significantly increased HDAC activity, administration to antibiotic-treated mice significantly decreased HDAC activity to the levels observed in SPF mice (Figure 2D). Similarly, the levels of acetylated histones in *CAPZA1* proximal promoter region were significantly decreased by antibiotic administration, and administration of But to antibiotic-treated mice significantly increased histone acetylation to the same levels as that in SPF mice (Figure 2E). These findings suggest that several genes expressed in the gastric epithelium, including *CAPZA1*, are regulated by histone acetylation induced by gastric microbiota-derived SCFAs. Next, we examined whether the generation of CD44v9-positive cells was enhanced in the SCFA-treated mice infected with *H pylori*. In the antibiotic-treated mice, only a few CD44v9 immunostaining signals were detected, and CD44v9-positive staining was confined to the pit area of both the gastric corpus and antrum (Figure 2F). In contrast, CD44v9 immunostaining signals of antibiotic-treated mice were increased by the administration of Pro or But, and CD44v9-positive staining was detected in both the pit and base regions of the corpus and antrum (Figure 2F). Our findings highlight the contribution of SCFAs to CD44v9-positive gastric epithelial cell generation by upregulating CAPZA1 expression during *H pylori* infection.

SCFA-Induced CD44v9-Positive Cell Development Also Detected in H pylori*–Infected Gastric Organoids Models*

To analyze normal epithelial cell responses leading to CD44v9-positive cell generation by SCFAs under *H pylori* infection, we developed gastric organoids from murine gastric corpus and antrum and then transferred the developed organoid cultures to a 2D monolayer culture using transwell inserts (Figure 3A). The resulting 2D-monolayer culture, named mucosoid, recapitulated the gastric mucosa and represented normal stem cell–driven cultures (Figure 3A).[10] In gastric corpus-derived mucosoids, translocated CagA was detected only during *H pylori* infection in the presence of But. CD44v expression was enhanced in association with increased CAPZA1 expression (Figure 3B). In gastric antrum-derived mucosoids, translocated CagA was detected following *H pylori* infection, and CD44v expression was further enhanced by treatment with But (Figure 3B). These results suggested that the gastric corpus and antrum differed in their responsiveness to *H pylori*–mediated CagA injection into epithelial cells. In addition, during immunostaining analysis for CAPZA1 and CD44v9 during *H pylori* infection in the presence of Pro or But, CD44v9-positive cells were observed within strong CAPZA1 staining in both corpus- and antrum-derived mucosoids (Figure 3C). In contrast, infection with the *H pylori* Δ*cag*PAI strains under the same conditions resulted in no detectable CD44v9-positive cells, despite strong CAPZA1 staining (Figure 3C). CD44v9-positive cells were barely detectable in the *H pylori*–infected mucosoids in the absence of But (Figure 4D). In contrast, *H pylori* infection under presence of But significantly increased CD44v9-positive cell populations in both corpus and antrum-derived mucosoids within strong CAPZA1 staining, but not *H pylori* Δ*cag*PAI infection (Figure 3D and E). These results indicate that CD44v9-positive cell generation is promoted by intracellular CagA accumulation, which is mediated by SCFA-induced CAPZA1 overexpression.

CD44v9-Positive Cells Induced by CagA Accumulation in the Presence of but Exhibited Stem Cell–like Properties Exclusively in Antrum-Derived Mucosoid

To determine whether CagA-induced CD44v9-positive cells exhibited stem cell–like characteristics, we assessed the expression of LGR5, KLF5, and SALL4, which are gastric stem cell markers using immunostaining. In the presence of But, stem cell marker expression was detected in CD44v9-positive cells, but not in those infected with the *H pylori* Δ*cag*PAI strain, suggesting that stem cell-like properties

Figure 1. SCFAs induce CAPZA1 expression in AGS cells and promote CagA accumulation and CD44v expression under *H pylori* infection. (A) Western blotting analysis of CAPZA1 expression in AGS cells treated with propionate (Pro) or butyrate (But), with signal quantification using ImageJ software. (B) HDAC activity in AGS cells treated with Pro or But. Statistical analysis was performed using one-way ANOVA followed by Tukey's test. *$P < .05$, **$P < .01$. (C) ChIP assay showing histone acetylation in the CAPZA1 promoter region of AGS cells treated with Pro or But. Chromatin was immunoprecipitated with anti-histone H3 (acetyl K9) or control IgG, and enrichment of CAPZA1 promoter regions was assessed using real-time PCR. (D) Immunofluorescence analysis of autolysosome formation in But-treated AGS cells infected with *H pylori* ATCC 700392. Lysosomes and nuclei were stained with LysoTracker and 4',6-diamidino-2-phenylindole, respectively. Scale bar = 50 μm. (E) Western blotting analysis of CagA accumulation in But-treated AGS cells infected with *H pylori* ATCC 700392. (F) Western blotting analysis of CD44v expression in Pro- or But-treated AGS cells infected with *H pylori* G27 or G27 Δ*cag*PAI strains.

were conferred by CagA (Figure 4). Notably, CD44v9-positive cells were detected in *H. pylori*–infected corpus-derived mucosoids in the presence of But; however, stem cell marker expression was not observed regardless of CagA-positive *H pylori* infection and But treatment (Figure 4). These results suggest that the mechanisms underlying cancer stem cell development differ between the gastric corpus and antrum, and that epithelial cellular responses to CagA also vary between these regions. We also stained for TFF2, a marker of spasmolytic polypeptide-expressing metaplasia (SPEM) cells. SPEM cells arise from cryptic progenitor cells located at the base of gastric glands.[28,29] TFF2 staining was not detected in *H pylori*–infected corpus-derived mucosoids treated with

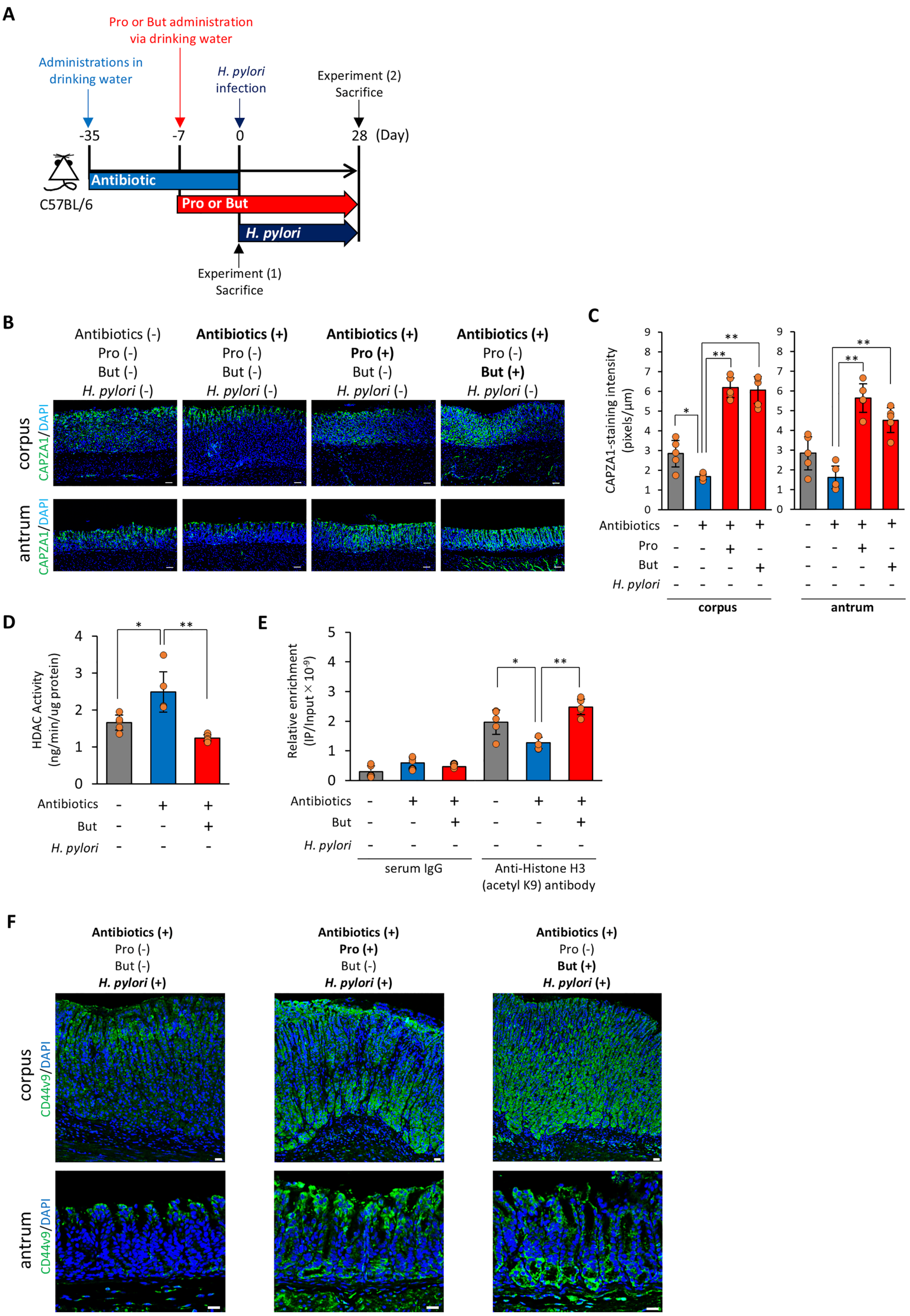

But (Figure 4). Similarly, in the antrum-derived mucosoids, TFF2 staining was barely detectable in CD44v9-positive cells (Figure 4). These results indicated that CagA-induced CD44v9-positive cells in antrum-derived mucosoids do not exhibit SPEM cell properties. Collectively, our findings using gastric mucosoids support a model in which SCFAs, through epigenetic modulation of CAPZA1, promote intracellular CagA accumulation, thereby triggering CD44v9-positive cell generation. In the antrum, CagA-induced CD44v9-positive cells exhibited stem cell–like properties distinct from those of SPEM cells, suggesting that they may represent the origin of GC stem cells.

Altered SCFAs Concentrations Linked to Gastric Microbiota Shifts May Contribute to Early GC Development in H pylori*–Infected Individuals*

To investigate the association between gastric SCFAs concentrations and GC development in *H. pylori*–infected patients, human gastric juice samples were collected, and SCFA levels and gastric microbiota compositions were compared between patients with and without GC. Inclusion and study flow diagrams are presented in Figure A1. A total of 66 participants (44 male and 22 female) were enrolled in the study. Microbiome analysis of the gastric juice was performed in 32 individuals, excluding 25 uninfected individuals with *H pylori* and nine individuals for whom insufficient bacterial DNA in the gastric juice could not be collected. The baseline clinical characteristics of the subjects were analyzed in this study (Tabel 1). The average age of *H pylori* (+) group (n = 41) was older than that in the *H pylori* (−) group (n = 25). The mean body mass index (BMI) and sex were not significantly different between the 2 groups. In the *H pylori* (+) group, 10 patients had a current infection, and 31 had a history of previous eradication. The number of patients taking anti-acid medications (proton pump inhibitors/potassium-competitive acid blockers) did not differ between the 2 groups. We analyzed the *H pylori* (+) group divided by the presence of GC because there was no GC patient in the *H pylori* (−) group. The *H pylori* (+) GC (+) group had a significantly lower BMI than the *H pylori* (+) GC (−) group, but there were no differences in age, sex, or proton pump inhibitor/potassium-competitive acid blocker usage rates (Table A1). To further examine whether these SCFA concentrations were correlated with the development of GC, we analyzed the concentration of SCFAs in human subjects based on the presence of *H pylori* infection. Pro and But levels were higher in the *H pylori* (+) group, with the former showing statistical significance (Figure 5A). Pro and But tended to be higher in the group with GC than those in the group without GC (Figure 5B). Next, we analyzed the gastric microbiome of *H pylori* (+) subjects to examine the types of bacteria responsible for the differences in SCFA levels (Figure 5C). Gastric juice samples were isolated, and the microbial composition was assessed using 16SrRNA sequencing.[30,31] The diversity of the gastric microbial composition tended to decrease in the group with GC compared with that in the group without GC (Figure 5C). Some bacterial taxa known to produce SCFAs, such as c_*Bacilli*, o_*Lactobacillales*, and p_*Firmicutes*, were enriched in the group with GC compared with those without GC (Figure 5D). To investigate the overall gastric microbiota composition of the differences in the production of SCFAs, analysis of β-diversity via a PCoA distribution 3D plot at the genus level revealed a clear division into two groups, A and B (Figure 5E). The participants with GC were distributed into both groups without bias (Figure 5E, red spots). PCoA indicated that the concentration of Pro or But derived from the composition of the gastric microbiota of group B tended to be higher than that of group A, but the differences were not significant (Figure 5F). Nevertheless, from a clinical perspective, despite no difference in the rates of atrophic gastritis or associated *H pylori* infection, all metaplasia of early GC was found in group B, whereas advanced GC was significantly more frequent in group A than in group B (Figure 5G). *H pylori* tends to diminish as GC progresses, accompanied by dynamic shifts in the gastric microbiota.[1] These alterations are thought to influence gastric SCFAs. Our findings suggest that diverse factors dependent on the composition of the gastric microbiota are involved in GC development in *H pylori*–infected mucosa, and that increased SCFA levels may represent one of the contribution to this process.

Discussion

SCFAs act as endogenous inhibitors of class I and II HDACs, thereby influencing epigenetic regulation.[24,25] These histone modifications have been implicated in a wide

Figure 2. *In vivo* SCFA-induced CD44v9 expression analysis in *H pylori*–infected gastric mucosa. (A) Schematic of mouse model used to evaluate the effects of SCFAs during *H pylori* infection. Endogenous SCFAs were depleted by administering antibiotics (ampicillin, metronidazole, neomycin [1 g/L each], and vancomycin [0.5 g/L]) in drinking water for 4 weeks, followed by administration of 300 mM Pro or But for 1 week before *H pylori* infection. (B, C) Immunohistochemical analysis (B) and quantification (C) of CAPZA1 expression in the gastric corpus and antrum after Pro or But treatment in the absence of *H pylori* infection. Scale bar = 50 μm. ImageJ was used for quantification. Statistical analysis was performed using one-way ANOVA followed by Tukey's test. *$P < .05$, **$P < .01$. (D) Measurement of HDAC activity in gastric mucosa following But treatment. Statistical analysis was performed using one-way ANOVA followed by Tukey's test. *$P < .05$, **$P < .01$. (E) *In vivo* ChIP assay of histone acetylation in the CAPZA1 promoter region in But-treated gastric mucosa. Chromatin was immunoprecipitated using anti-histone H3 (acetyl K9) or control IgG, followed by real-time PCR. Statistical analysis was performed using one-way ANOVA followed by Tukey's test. *$P < .05$, **$P < .01$. (F) Immunohistochemical analysis of CD44v9 expression in the gastric body and antrum of Pro- or But-treated mice infected with *H pylori*. Scale bar = 20 μm.

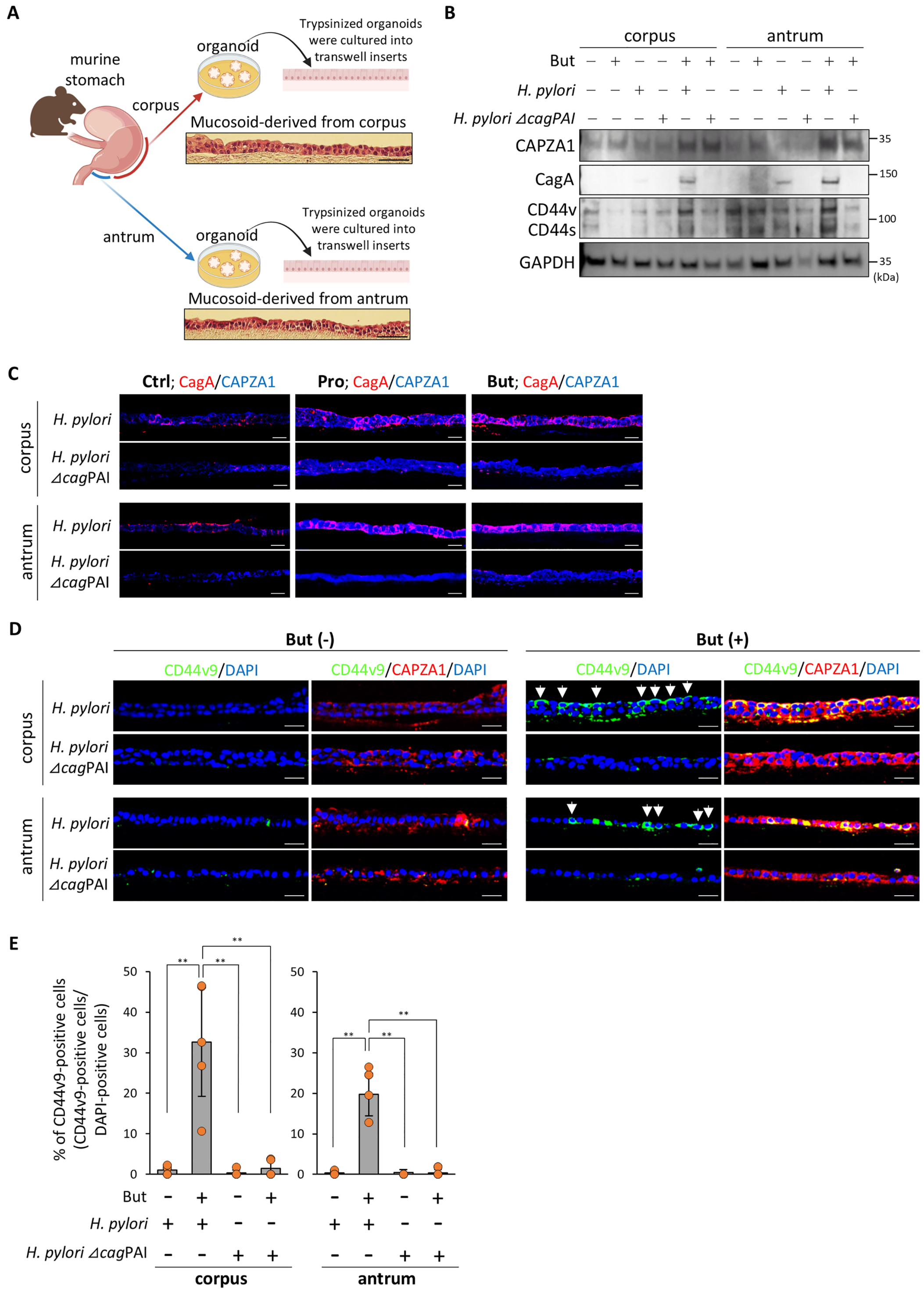

Figure 3. SCFA-induced CAPZA1 expression in mouse gastric organoids facilitates CagA accumulation and CD44v9-positive cell generation. (A) A schematic of organoid culture from the gastric corpus and antrum of mice, followed by trypsinization and seeding on Transwell inserts to generate mucosoids. Constructed mucosoids were subjected to hematoxylin and eosin (H&E) staining. (B) Changes in CD44s and CD44v expression in mucosoids derived from the gastric corpus and antrum following infection with *H pylori* G27 strains (CagA-positive *H pylori*) or *H pylori* G27 *cag*PAI-deletion mutant strains (Δ*cag*PAI) in the presence of But. (C) Stability of intracellular CagA in *H pylori* G27-infected mucosoids derived from the gastric corpus and antrum under Pro or But treatment. Scale bar = 20 μm. (D) Immunofluorescence analysis showing CD44v9-positive cell generation in mucosoids following infection with *H pylori* G27 strains (CagA-positive *H pylori*) or G27 *cag*PAI-deletion mutant strains (Δ*cag*PAI) in the presence of But. Scale bar = 20 μm. (E) Quantification of CD44v9^{+} cells induced by *H pylori* G27 strains (CagA-positive *H pylori*) or *H pylori* G27 *cag*PAI-deletion mutant strains (Δ*cag*PAI) infection in the presence of But. Statistical analysis was performed using one-way ANOVA followed by Tukey's test. $^{**}P < .01$.

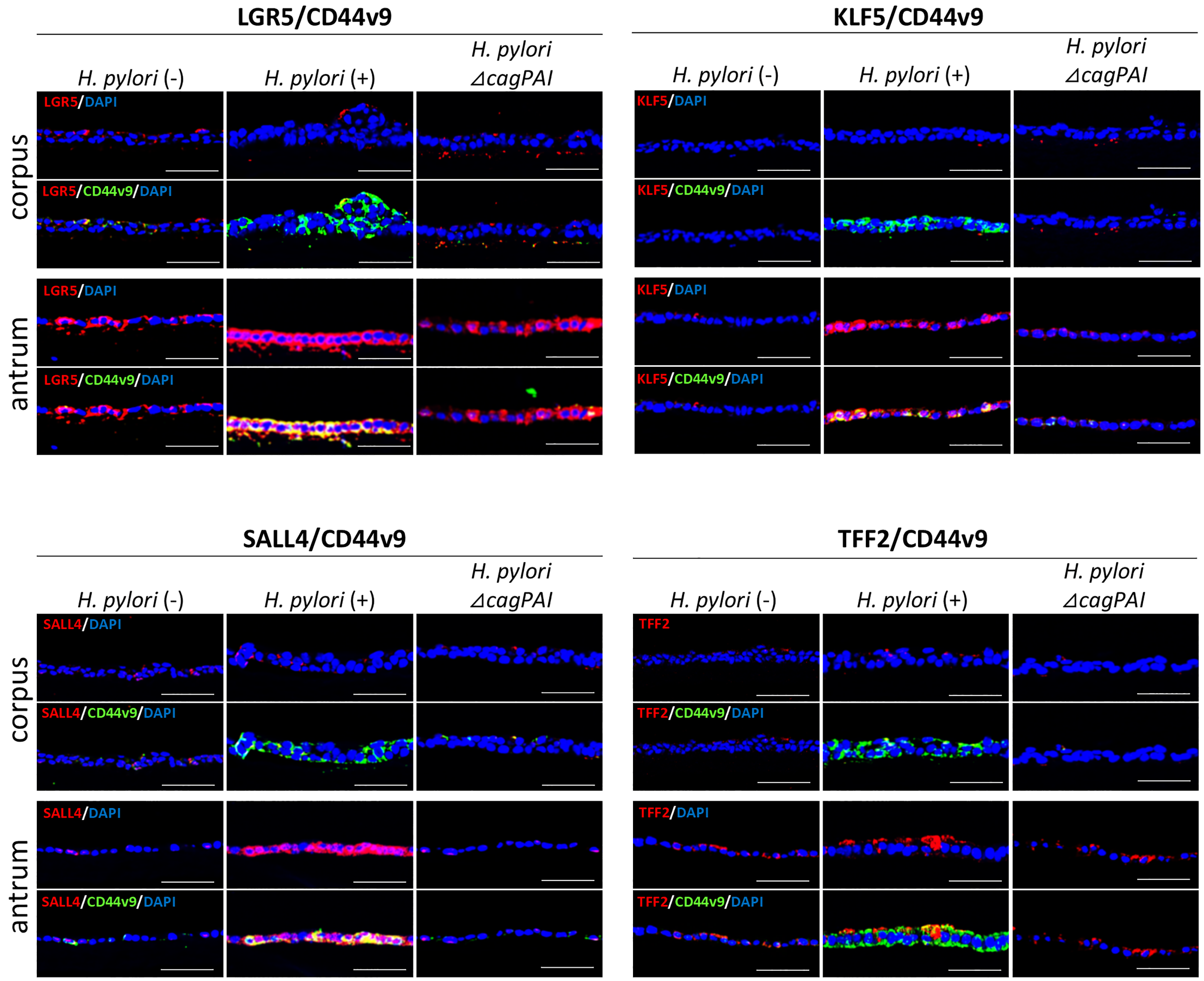

Figure 4. Stem cell characteristics of CD44v9-positive cells induced by *H pylori* G27 strains (CagA-positive *H pylori*) infection in the presence of butyrate (But). Immunofluorescence analysis of colocalization between CD44v9 and stem cell markers (LGR5, KLF5, and SALL4) in mucosoids derived from the gastric corpus and antrum of mice infected with *H pylori* G27 strains (CagA-positive *H pylori*) or *H pylori* G27 *cag*PAI-deletion mutant strains (Δ*cag*PAI) in the presence of But. Colocalization of CD44v9 and the SPEM marker TFF2 was also assessed. Scale bar = 50 μm.

range of cellular processes including differentiation, apoptosis, and tumor suppression. SCFAs activate multiple intracellular signaling cascades, largely through G protein-coupled receptors (GPCRs), notably free fatty acid receptors 2 and 3 (FFAR2/GPR43 and FFAR3/GPR41),[32,33] ultimately modulating gene expression via the activation of

Table 1. Baseline Clinical Characteristics of Human Participants

	H pylori (+) (n = 41)	*H pylori* (-) (n = 25)	*P* value
Age (mean y ± SD)	71.6 ± 12.6	60.4 ± 11.3	<.001
BMI (mean y ± SD)	22.7 ± 3.1	23.9 ± 2.9	N.S. (.11)
Men, n (%)	30 (73.1)	14 (56.0)	N.S. (.18)
Female, n (%)	11 (26.9)	11 (44.0)	
H pylori current infection, n (%)	10 (24.4)	0	
H pylori past infection, n (%)	31 (75.6)	0	
sPPI/P-CAB n, (%)	20 (48.7)	13 (53.0)	N.S. (>.99)

BMI, body mass index; PPI/P-CAB, proton pump inhibitor/potassium-competitive acid blocker.

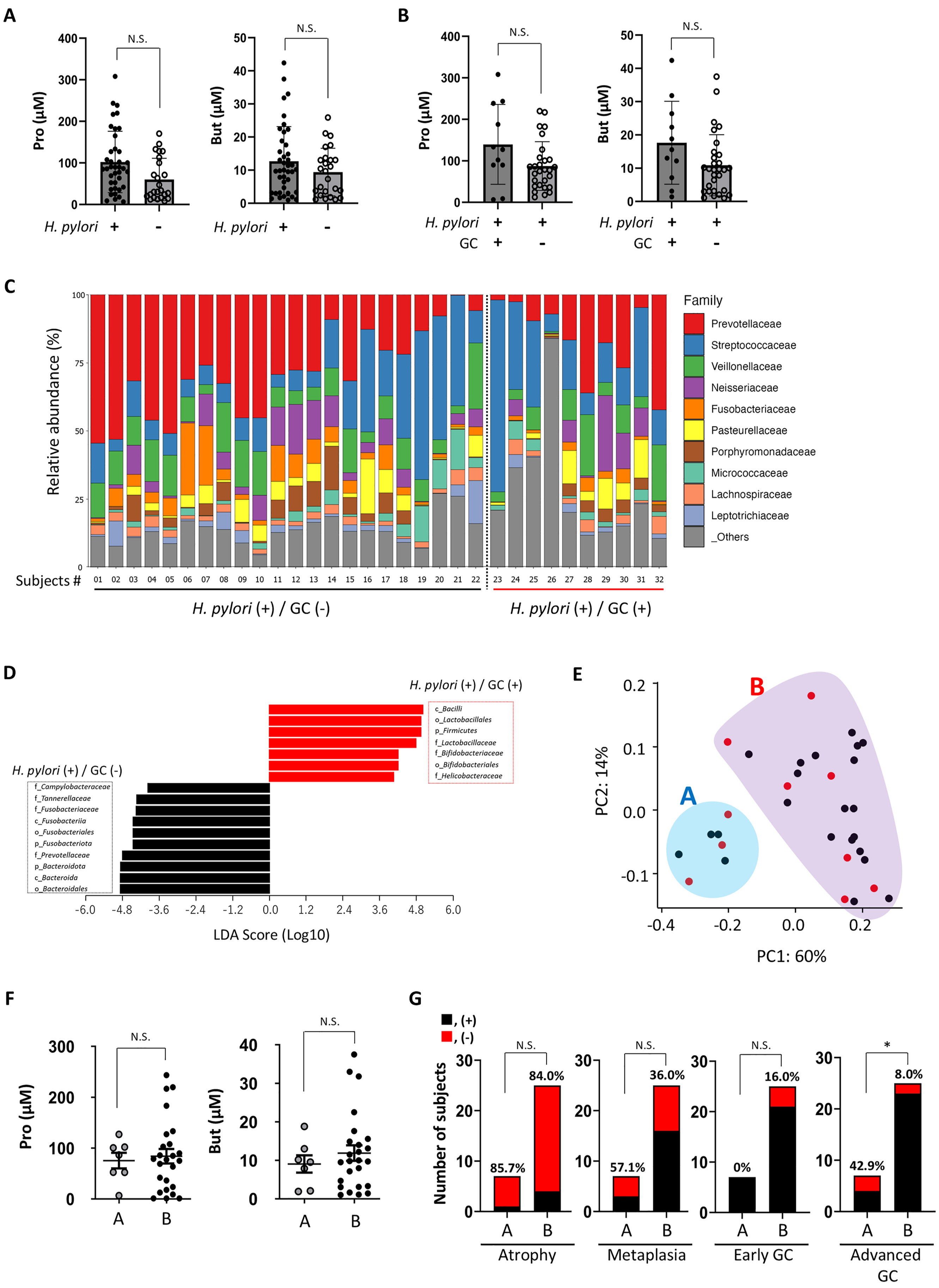
A
Pro (μM)
N.S.
H. pylori + -
But (μM)
B
H. pylori + +
GC + -
C
Relative abundance (%)
Family
Prevotellaceae
Streptococcaceae
Veillonellaceae
Neisseriaceae
Fusobacteriaceae
Pasteurellaceae
Porphyromonadaceae
Micrococcaceae
Lachnospiraceae
Leptotrichiaceae
_Others
Subjects #
H. pylori (+) / GC (-)
H. pylori (+) / GC (+)
D
c_Bacilli
o_Lactobacillales
p_Firmicutes
f_Lactobacillaceae
f_Bifidobacteriaceae
o_Bifidobacteriales
f_Helicobacteraceae
f_Campylobacteraceae
f_Tannerellaceae
f_Fusobacteriaceae
c_Fusobacteriia
o_Fusobacteriales
p_Fusobacteriota
f_Prevotellaceae
p_Bacteroidota
c_Bacteroida
o_Bacteroidales
LDA Score (Log10)
E
PC2: 14%
PC1: 60%
F
G
■, (+)
■, (-)
Number of subjects
85.7%
84.0%
57.1%
36.0%
0%
16.0%
42.9%
8.0%
*
Atrophy
Metaplasia
Early GC
Advanced GC

various transcription factors. SCFA-mediated epigenetic effects can occur either indirectly through GPCR signaling or directly via cellular uptake through monocarboxylate transporters.[24,25] In this study, we showed that SCFAs upregulate CAPZA1 expression in gastric mucosal epithelial cells via their HDAC inhibitory activity. The resulting CAPZA1-overexpressing epithelial cells were exploited by *H pylori* as scaffold cells for the stabilization of CagA. Stabilized CagA subsequently enhances CD44v9 expression, which is associated with stem cell differentiation, and may contribute to the early stages of gastric carcinogenesis. Our findings suggest that SCFA-induced CAPZA1-overexpressing epithelial cells constitute a permissive niche that amplifies *H pylori*–driven oncogenic signaling. However, important questions remain regarding the tissue- and cell type-specific contexts in which SCFAs exert their HDAC-inhibitory effects as well as the biological significance of CAPZA1-overexpressing epithelial cells in maintaining gastric epithelial homeostasis. Further investigations are warranted to elucidate the molecular mechanisms underlying SCFA-induced CAPZA1 upregulation, particularly the relative contribution of GPCR-mediated signaling to monocarboxylate transporter–dependent intracellular uptake.

Mechanistically, CagA promotes stemness via multiple pathways, including nuclear export of the transcription factor FOXO3a via the PI3K/Akt pathway, and upregulation of pluripotency-associated transcription factors, such as Nanog and Oct4 via Wnt/β-catenin signaling pathway activation.[34,35] Using gastric antrum-derived mucosoids infected with *H pylori*, we observed robust expression of stemness markers (LGR5, KLF5, and SALL4) in CD44v9-positive epithelial cells (Figure 4). In contrast, these markers were not detected in *H pylori* ΔCagA-infected mucosoids (Figure 4). These findings suggest that the stemness phenotype of CD44v9-positive gastric epithelial cells is induced by stabilized CagA. In contrast, in gastric corpus-derived mucosoids infected with *H pylori*, the expression of these stemness markers in CD44v9-positive cells was barely detectable (Figure 4), suggesting region-specific differences in epithelial cell responses to CagA. These differences may reflect variations in the modes of CagA translocation, receptor expression, or intracellular signaling competence. Notably, these findings align with the clinical observations reported by Kim *et al.*, who demonstrated that early GC most frequently arises in the antrum, particularly along the lesser curvature, and exhibits distinctive locational characteristics.[36] Our data provide mechanistic insights into these clinical patterns, suggesting that regional differences in epithelial susceptibility to CagA-mediated signaling contribute to site-specific gastric carcinogenesis.

SCFAs influence epithelial cell behavior, including proliferation, differentiation, and inflammatory responses.[8] In this study, we focused on the induction of CAPZA1 overexpression in normal epithelial cells via HDAC-inhibitory activity of SCFAs. Using normal murine gastric epithelial cell–derived mucosoids, we showed that *H pylori* infection in the presence of SCFAs led to the emergence of CD44v9-positive cancer stem-like cells (Figures 3 and 4). Our previous findings demonstrated that CD44v9-positive cell generation in *H pylori*–infected gastric mucosa significantly increased the risk of metachronous recurrence following endoscopic resection of early GC.[3] Thus, the emergence of CD44v9 in *H pylori*–infected gastric mucosa via CAPZA1 expression induction by SCFAs is thought to be a precancerous change in normal gastric epithelial cells. Studies using normal murine gastric epithelial cell–derived mucosoids provide valuable insights into the signal transduction pathways that induce precancerous changes. In contrast, GC-derived mucosoids may be suitable tools for research focusing on the effects of SCFAs on GC progression, prognosis, and metastasis. Although further investigation using cancer-derived mucosoids is needed to understand the role of SCFAs in GC progression, prognosis, and recurrence, the current findings demonstrate the developmental mechanisms of CD44v9-positive cancer stem-like cells through the cooperative action of *H pylori* infection and SCFAs in murine normal gastric epithelial cell–derived mucosoids.

Once delivered into host cells, CagA becomes tyrosine-phosphorylated at conserved EPIYA (Glu-Pro-Ile-Tyr-Ala) motifs, thereby initiating oncogenic signaling cascades.[37,38] Thus, the type IV secretion system (T4SS)-mediated injection of CagA is considered a pivotal event in *H pylori*–associated gastric carcinogenesis. Three distinct routes of CagA delivery into gastric epithelial cells have been described: CagA can be injected through direct

Figure 5. The gastric concentrations of SCFAs in human subjects and analysis of gastric microbiota. (A) The concentrations of propionate and butyrate in gastric juice, divided by the presence or absence of *H pylori* infection, were measured. Mann–Whitney U test, N.S.: not significant; *$P < .05$. (B) The concentrations of propionate and butyrate in the gastric juice divided by the presence or absence of gastric cancer among *H. pylori*–infected subjects were measured. Mann–Whitney U test; N.S., not significant. (C) Gastric microbiome characterization. The microbial composition of the gastric juice divided by the presence or absence of gastric cancer among *H pylori*–infected subjects was analyzed using 16S rRNA sequencing. The relative abundances of bacterial families are shown. (D) Differences in bacterial families among *H pylori*–infected subjects, divided by the presence or absence of gastric cancer, were analyzed using linear discriminant analysis effect size method. (E) The overall gastric microbiota was analyzed by β-diversity via PCoA distribution 3D plot at the genus level revealed a clear division into two groups, A and B (red spots; subjects with gastric cancer, black spots; subjects without gastric cancer). (F) The concentrations of propionate (Pro) and butyrate (But) in the gastric juice divided by groups A and B according to PCoA distribution were measured. Mann–Whitney U test; N.S., not significant. (G) Comparison of clinical findings in groups A and B according to PCoA distribution. Chi-square test. N.S., not significant.

binding of the T4SS to integrin $\beta1$[39]; alternatively, CagA may be internalized via interaction with externalized phosphatidylserine, forming a phosphatidylserine–CagA complex that is subsequently taken up by host cells[9]; and finally, the outer membrane protein HopQ of *H pylori* can engage carcinoembryonic antigen-related cell adhesion molecules, facilitating CagA translocation.[40,41] Notably, in CRISPR/Cas9-modified AGS and KatoIII cells, even integrin-deficient cells retain the ability to translocate CagA, suggesting that integrin-independent mechanisms contribute to uptake.[42] Moreover, studies using duodenal AZ521 cells indicate that while HopQ–carcinoembryonic antigen-related cell adhesion molecule engagement is sufficient for CagA internalization, tyrosine phosphorylation of CagA requires T4SS interaction with integrin $\beta1$.[43] These findings underscore the cell-type-dependent variation in CagA delivery mechanisms and imply that such variations contribute to differences in the intracellular activity of CagA. Further investigation is warranted to determine whether distinct CagA translocation routes are preferentially utilized in gastric antral versus corpus epithelial cells and how such differences influence the acquisition of stemness traits in CD44v9-positive cells and their tumorigenic potential. Our findings provide novel insights into the site-specific mechanisms of oncogenesis and may inform the development of compartment-targeted molecular therapeutics and early diagnostic biomarkers for *H pylori*–associated GC.

SPEM is characterized by TFF2 and zymogenic chief cell marker expression, which typically arise in regions of intestinal metaplasia, where gastric epithelial cells acquire intestinal-like features in the context of *H pylori* infection.[28,29] While the cellular origin of SPEM remains unclear, two major hypotheses exist: (1) transdifferentiation or dedifferentiation from mature chief cells and (2) metaplastic transformation arising from stem or progenitor cells through genetic or epigenetic alterations. In this study, immunostaining for TFF2, a canonical SPEM marker, in gastric antrum-derived mucosoid models revealed limited TFF2 expression overall, and notably, minimal colocalization with CD44v9-positive cells, a population dependent on CagA accumulation (Figure 4). These results suggested that SPEM is unlikely to arise from mature epithelial cells undergoing CagA-driven transformation. Rather, they support a model in which SPEM originates from epigenetically reprogrammed stem or progenitor cells, independent of direct CagA influence. CagA promotes stem-like phenotypes in gastric epithelial cells by dysregulating β-catenin signaling or activating oncogenic pathways such as YAP.[44,45] Consistent with these findings, our results indicated that SCFAs create a permissive niche that facilitates CagA accumulation and function via CAPZA1-overexpressing cell induction, thereby promoting the reprogramming of gastric epithelial cells into a stem-like state. Collectively, our data suggest that CD44v9-positive cancer stem-like cells emerge via CagA-dependent transdifferentiation or dedifferentiation of mature epithelial cells rather than through pathways associated with SPEM. Furthermore, although CagA accumulation was observed in the gastric corpus, it did not result in detectable dedifferentiation signaling, indicating a regional restriction in epithelial responsiveness to CagA-induced stemness.

Studies using insulin-gastrin transgenic mice have demonstrated that specific gastric commensal bacteria are required for *H pylori*–associated GC development.[2] *H pylori* infection significantly alters the composition of the gastric microbiota,[46–48] and the microbial profiles in patients with GC differ markedly from those in patients with chronic gastritis.[31] These reports indicate that certain commensal gastric bacteria may synergize with *H pylori* to promote gastric tumorigenesis. However, to date, no definitive reports have identified the specific bacterial taxa that cooperate with *H pylori* to accelerate gastric carcinogenesis. This study provides insights into the enrichment of the gastric microbiota composition of *H pylori*–infected stomachs by SCFA-producing bacteria, which increases the risk of gastric carcinogenesis. This highlights the importance of elucidating the gastric environmental factors that favor the colonization or expansion of SCFA-producing bacteria in the stomach. A decrease in the gastric luminal pH confers a competitive advantage to But-producing bacteria over Pro-producing species. Moreover, under acidic conditions, commensal bacterial metabolism appears to favor But synthesis over the production of alternative metabolic byproducts.[49–51] Although further clinical investigations are needed to understand the relationship between *H pylori* infection–induced pH changes and GC risk, our findings suggest that the modulation of SCFA-producing bacterial populations and the identification of host or microbial factors that drive this enrichment are critical for understanding the mechanisms underlying the transition from *H pylori* infection to gastric tumorigenesis.

Although gastric juice–derived microbiota may include transient microorganisms introduced via food ingestion or oral contamination, in this study, microbiome analysis was performed on gastric juice samples rather than on mucosal biopsies based on several scientific and practical considerations. This study aimed to investigate the role of SCFAs in *H pylori*–induced gastric carcinogenesis. SCFA concentrations in the gastric lumen are influenced by microbial composition and individual dietary patterns. Filippo *et al.* reported that fiber-rich diets promote SCFA-producing bacteria in the gut lumen, suggesting that the microbiota derived from gastric juice, which is more sensitive to dietary influences, may better reflect the SCFA-producing potential than the mucosal biopsy-derived microbiota.[52] In addition, gastric juice sampling is noninvasive and easily performed, posing a minimal burden to patients, particularly older individuals or those with chronic conditions, compared to mucosal biopsies. This makes gastric juice–derived microbiome analysis a more feasible approach for developing clinically applicable tools for rapid and accessible GC risk assessment. Gastric juice microbiota exhibits greater microbial diversity than mucosal biopsies in patients with *H pylori*–negative GC.[53] Gastric juice

microbiome profiling can predict GC occurrence and progression with high accuracy, strongly highlighting its potential as a noninvasive diagnostic tool.[30,54] Moreover, unlike mucosal biopsies, which reflect localized microbial communities, gastric juice offers a broader view of microbial populations across the stomach, from the corpus to the antrum. In our study, we identified an association between SCFA-producing bacteria in gastric juice and the emergence of CD44v9-positive GC stem cells, providing a specific rationale for their use in gastric carcinogenesis risk assessment. In addition, we acknowledge the importance of determining the optimal conditions for sample collection. Gastric fluid samples were collected during routine gastrointestinal endoscopy (without any emergency procedures). As all patients were instructed to fast and refrain from fluid intake from 9:00 p.m. on the day before the examination, the gastric fluid conditions were considered relatively standardized across participants. Collectively, these findings suggest that gastric juice microbiome analysis may provide meaningful insights into the microbial factors involved in gastric carcinogenesis.

In our study, even among *H pylori*–positive individuals, patients with GC exhibited a lower BMI than noncancer controls. Previous reports have also indicated that low BMI in GC patients is associated with poor prognosis and may exert immunosuppressive effects on postoperative adjuvant chemotherapy. Moreover, differences in intertumoral microbiota and alterations in metabolomic profiles have been demonstrated.[55] Taken together, these findings suggest that the elevated intragastric SCFA concentrations observed in GC patients in the present study may reflect tumor-promoting changes. Thus, intragastric SCFA levels could potentially serve as prognostic biomarkers in GC. Ultimately, targeting microbial metabolic dynamics or restoring healthy gastric microbial ecosystems may offer new preventive strategies against *H pylori*–associated GC.

Supplementary Materials

Material associated with this article can be found, in the online version, at https://doi.org/10.1016/j.gastha.2025.100860.

References

1. Suzuki H, Matsuzaki J. Gastric cancer: evidence boosts Helicobacter pylori eradication. Nat Rev Gastroenterol Hepatol 2018;15:458–460.
2. Lofgren JL, Whary MT, Ge Z, et al. Lack of commensal flora in helicobacter pylori-infected INS-GAS mice reduces gastritis and delays intraepithelial neoplasia. Gastroenterology 2011;140:210–220.
3. Hirata K, Suzuki H, Imaeda H, et al. CD44 variant 9 expression in primary early gastric cancer as a predictive marker for recurrence. Br J Cancer 2013;109:379–386.
4. Tsugawa H, Suzuki H, Saya H, et al. Reactive oxygen species-induced autophagic degradation of Helicobacter pylori CagA is specifically suppressed in cancer stem-like cells. Cell Host Microbe 2012;12:764–777.
5. Tsugawa H, Kato C, Mori H, et al. Cancer stem-cell marker CD44v9-Positive cells arise from helicobacter pylori-infected CAPZA1-Overexpressing cells. Cell Mol Gastroenterol Hepatol 2019;8:319–334.
6. Tsugawa H, Mori H, Matsuzaki J, et al. CAPZA1 determines the risk of gastric carcinogenesis by inhibiting Helicobacter pylori CagA-degraded autophagy. Autophagy 2019;15:242–258.
7. Steliou K, Boosalis MS, Perrine SP, et al. Butyrate histone deacetylase inhibitors. Biores Open Access 2012;1:192–198.
8. Mukhopadhya I, Louis P. Gut microbiota-derived short-chain fatty acids and their role in human health and disease. Nat Rev Microbiol 2025;23:635–651.
9. Murata-Kamiya N, Kikuchi K, Hayashi T, et al. Helicobacter pylori exploits host membrane phosphatidylserine for delivery, localization, and pathophysiological action of the CagA oncoprotein. Cell Host Microbe 2010;7:399–411.
10. Boccellato F, Woelffling S, Imai-Matsushima A, et al. Polarised epithelial monolayers of the gastric mucosa reveal insights into mucosal homeostasis and defence against infection. Gut 2019;68:400–413.
11. Rakoff-Nahoum S, Paglino J, Eslami-Varzaneh F, et al. Recognition of commensal microflora by toll-like receptors is required for intestinal homeostasis. Cell 2004;118:229–241.
12. Tsugawa H, Kabe Y, Kanai A, et al. Short-chain fatty acids bind to apoptosis-associated speck-like protein to activate inflammasome complex to prevent salmonella infection. PLoS Biol 2020;18:e3000813.
13. Sugano K, Tack J, Kuipers EJ, et al. Kyoto global consensus report on Helicobacter pylori gastritis. Gut 2015;64:1353–1367.
14. Japanese Gastric Cancer A. Japanese classification of gastric carcinoma: 3rd English edition. Gastric Cancer 2011;14:101–112.
15. Klindworth A, Pruesse E, Schweer T, et al. Evaluation of general 16S ribosomal RNA gene PCR primers for classical and next-generation sequencing-based diversity studies. Nucleic Acids Res 2013;41:e1.
16. Bolyen E, Rideout JR, Dillon MR, et al. Reproducible, interactive, scalable and extensible microbiome data science using QIIME 2. Nat Biotechnol 2019;37:852–857.
17. Bokulich NA, Kaehler BD, Rideout JR, et al. Optimizing taxonomic classification of marker-gene amplicon sequences with QIIME 2's q2-feature-classifier plugin. Microbiome 2018;6:90.
18. Pruesse E, Quast C, Knittel K, et al. SILVA: a comprehensive online resource for quality checked and aligned ribosomal RNA sequence data compatible with ARB. Nucleic Acids Res 2007;35:7188–7196.
19. Quast C, Pruesse E, Yilmaz P, et al. The SILVA ribosomal RNA gene database project: improved data processing and web-based tools. Nucleic Acids Res 2013;41:D590–D596.
20. Katoh K, Standley DM. MAFFT multiple sequence alignment software version 7: improvements in performance and usability. Mol Biol Evol 2013;30:772–780.
21. Price MN, Dehal PS, Arkin AP. FastTree 2–approximately maximum-likelihood trees for large alignments. PLoS One 2010;5:e9490.
22. McMurdie PJ, Holmes S. Phyloseq: an R package for reproducible interactive analysis and graphics of microbiome census data. PLoS One 2013;8:e61217.

23. Segata N, Izard J, Waldron L, et al. Metagenomic biomarker discovery and explanation. Genome Biol 2011;12:R60.
24. He J, Zhang P, Shen L, et al. Short-chain fatty acids and their association with signalling pathways in inflammation, glucose and lipid metabolism. Int J Mol Sci 2020;21:6356.
25. Rekha K, Venkidasamy B, Samynathan R, et al. Short-chain fatty acid: an updated review on signaling, metabolism, and therapeutic effects. Crit Rev Food Sci Nutr 2024;64:2461–2489.
26. Poh FJX, Shorey S. A literature review of factors influencing injurious falls. Clin Nurs Res 2020;29:141–148.
27. Miglioli-Galvao L, Pestana JOM, Santoro-Lopes G, et al. Severe Strongyloides stercoralis infection in kidney transplant recipients: a multicenter case-control study. PLoS Negl Trop Dis 2020;14:e0007998.
28. Petersen CP, Mills JC, Goldenring JR. Murine models of gastric corpus preneoplasia. Cell Mol Gastroenterol Hepatol 2017;3:11–26.
29. Schmidt PH, Lee JR, Joshi V, et al. Identification of a metaplastic cell lineage associated with human gastric adenocarcinoma. Lab Invest 1999;79:639–646.
30. Zeng R, Gou H, Lau HCH, et al. Stomach microbiota in gastric cancer development and clinical implications. Gut 2024;73:2062–2073.
31. Ferreira RM, Pereira-Marques J, Pinto-Ribeiro I, et al. Gastric microbial community profiling reveals a dysbiotic cancer-associated microbiota. Gut 2018;67:226–236.
32. Priyadarshini M, Kotlo KU, Dudeja PK, et al. Role of short chain fatty acid receptors in intestinal physiology and pathophysiology. Compr Physiol 2018;8:1091–1115.
33. Barki N, Jenkins L, Marsango S, et al. Phosphorylation bar-coding of free fatty acid receptor 2 is generated in a tissue-specific manner. Elife 2023;12:RP91861.
34. Chen ZW, Dong ZB, Xiang HT, et al. Helicobacter pylori CagA protein induces gastric cancer stem cell-like properties through the Akt/FOXO3a axis. J Cell Biochem 2024;125:e30527.
35. Yong X, Tang B, Xiao YF, et al. Helicobacter pylori upregulates nanog and Oct4 via Wnt/beta-catenin signaling pathway to promote cancer stem cell-like properties in human gastric cancer. Cancer Lett 2016;374:292–303.
36. Kim K, Cho Y, Sohn JH, et al. Clinicopathologic characteristics of early gastric cancer according to specific intragastric location. BMC Gastroenterol 2019;19:24.
37. Hatakeyama M, Higashi H. Helicobacter pylori CagA: a new paradigm for bacterial carcinogenesis. Cancer Sci 2005;96:835–843.
38. Hatakeyama M. Oncogenic mechanisms of the Helicobacter pylori CagA protein. Nat Rev Cancer 2004;4:688–694.
39. Kaplan-Turkoz B, Jimenez-Soto LF, Dian C, et al. Structural insights into Helicobacter pylori oncoprotein CagA interaction with beta1 integrin. Proc Natl Acad Sci USA 2012;109:14640–14645.
40. Koniger V, Holsten L, Harrison U, et al. Helicobacter pylori exploits human CEACAMs via HopQ for adherence and translocation of CagA. Nat Microbiol 2016;2:16188.
41. Javaheri A, Kruse T, Moonens K, et al. Helicobacter pylori adhesin HopQ engages in a virulence-enhancing interaction with human CEACAMs. Nat Microbiol 2016;2:16189.
42. Zhao Q, Busch B, Jimenez-Soto LF, et al. Integrin but not CEACAM receptors are dispensable for Helicobacter pylori CagA translocation. Plos Pathog 2018;14:e1007359.
43. Nakano M, Yahiro K, Yamasaki E, et al. Helicobacter pylori VacA, acting through receptor protein tyrosine phosphatase alpha, is crucial for CagA phosphorylation in human duodenum carcinoma cell line AZ-521. Dis Model Mech 2016;9:1473–1481.
44. Murata-Kamiya N, Kurashima Y, Teishikata Y, et al. Helicobacter pylori CagA interacts with E-cadherin and deregulates the beta-catenin signal that promotes intestinal transdifferentiation in gastric epithelial cells. Oncogene 2007;26:4617–4626.
45. Li N, Feng Y, Hu Y, et al. Helicobacter pylori CagA promotes epithelial mesenchymal transition in gastric carcinogenesis via triggering oncogenic YAP pathway. J Exp Clin Cancer Res 2018;37:280.
46. Maldonado-Contreras A, Goldfarb KC, Godoy-Vitorino F, et al. Structure of the human gastric bacterial community in relation to Helicobacter pylori status. ISME J 2011;5:574–579.
47. Jo HJ, Kim J, Kim N, et al. Analysis of gastric microbiota by pyrosequencing: minor role of bacteria other than Helicobacter pylori in the gastric carcinogenesis. Helicobacter 2016;21:364–374.
48. Schulz C, Schutte K, Koch N, et al. The active bacterial assemblages of the upper GI tract in individuals with and without helicobacter infection. Gut 2018;67:216–225.
49. Louis P, Flint HJ. Formation of propionate and butyrate by the human colonic microbiota. Environ Microbiol 2017;19:29–41.
50. Walker AW, Duncan SH, McWilliam Leitch EC, et al. pH and peptide supply can radically alter bacterial populations and short-chain fatty acid ratios within microbial communities from the human colon. Appl Environ Microbiol 2005;71:3692–3700.
51. Duncan SH, Louis P, Thomson JM, et al. The role of pH in determining the species composition of the human colonic microbiota. Environ Microbiol 2009;11:2112–2122.
52. De Filippo C, Cavalieri D, Di Paola M, et al. Impact of diet in shaping gut microbiota revealed by a comparative study in children from Europe and rural Africa. Proc Natl Acad Sci USA 2010;107:14691–14696.
53. Sun QH, Zhang J, Shi YY, et al. Microbiome changes in the gastric mucosa and gastric juice in different histological stages of helicobacter pylori-negative gastric cancers. World J Gastroenterol 2022;28:365–380.
54. Wei Q, Zhang Q, Wu Y, et al. Analysis of bacterial diversity and community structure in gastric juice of patients with advanced gastric cancer. Discov Oncol 2023;14:7.
55. Liu K, Jiang Z, Ma Y, et al. Multiomics insights into BMI-related intratumoral microbiota in gastric cancer. Front Cell Infect Microbiol 2025;15:1511900.

Received July 7, 2025. Accepted December 4, 2025.

Correspondence:
Address correspondence to: Hitoshi Tsugawa, PhD, Transkingdom Signaling Research Unit, Division of Host Defense Mechanism, Tokai University School of Medicine, 143 Shimokasuya, Isehara, Kanagawa 259-1193, Japan. e-mail: tsugawa.hitoshi.r@tokai.ac.jp; or Hidekazu Suzuki, MD, PhD, Division of Gastroenterology, Department of Internal Medicine, Tokai University School of Medicine, 143 Shimokasuya, Isehara, Kanagawa 259-1193, Japan. e-mail: hsuzuki@tokai.ac.jp.

Acknowledgments:
The authors are grateful to the Support Center for Medical Research and Education at the Tokai University School of Medicine for technical assistance.

Authors' Contributions:
Hitoshi Tsugawa: Conceptualization, methodology and data acquisition, investigation, visualization, funding acquisition, project administration, writing – original draft, writing – review and editing. Jin Imai: Methodology and data acquisition, investigation, visualization, writing – original draft, writing – review and editing. Hidekazu Suzuki: project administration and writing – review and editing. Eiji Sugiyama: Investigation. Chanudporn Sugiyama: Investigation. Takashi Ueda: Investigation. Miwa Hirai: Investigation. Kenichiro Todoroki: Investigation.

Conflicts of Interest:
Hitoshi Tsugawa received funding for joint research from Taiyo Kagaku Co., Ltd. and Tsumura Corporation. The funding agencies played no role in the study design, data collection and analysis, decision to publish, or manuscript preparation. The remaining authors disclose no conflicts.

Funding:
This study was supported by a Grant-in-Aid for Scientific Research (B) (25K02412, to H.T.), Grant-in-Aid for Scientific Research (C) (22K06585, to H.T.), the Vehicle Racing Commemorative Foundation (6449, to H.T.), and the Yakult Bioscience Research Foundation (to H.T.). The funders had no role in the study design, data collection and analysis, decision to publish, or manuscript preparation.

Data Transparency:
The main data supporting the findings of this study, including all raw western blot images and source data, will be made publicly available in the article and Supplementary Information.

Reporting Guidelines:
Not applicable.

Gastro Hep Advances, Volume *5*

Supplemental information

Helicobacter pylori Exploit Short-Chain Fatty Acids-Induced CAPZA1 Overexpression to Emerge CD44v9-Positive Stemness

Hitoshi Tsugawa, Jin Imai, Eiji Sugiyama, Chanudporn Sugiyama, Takashi Ueda, Miwa Hirai, Kenichiro Todoroki, and Hidekazu Suzuki

Supplementary information

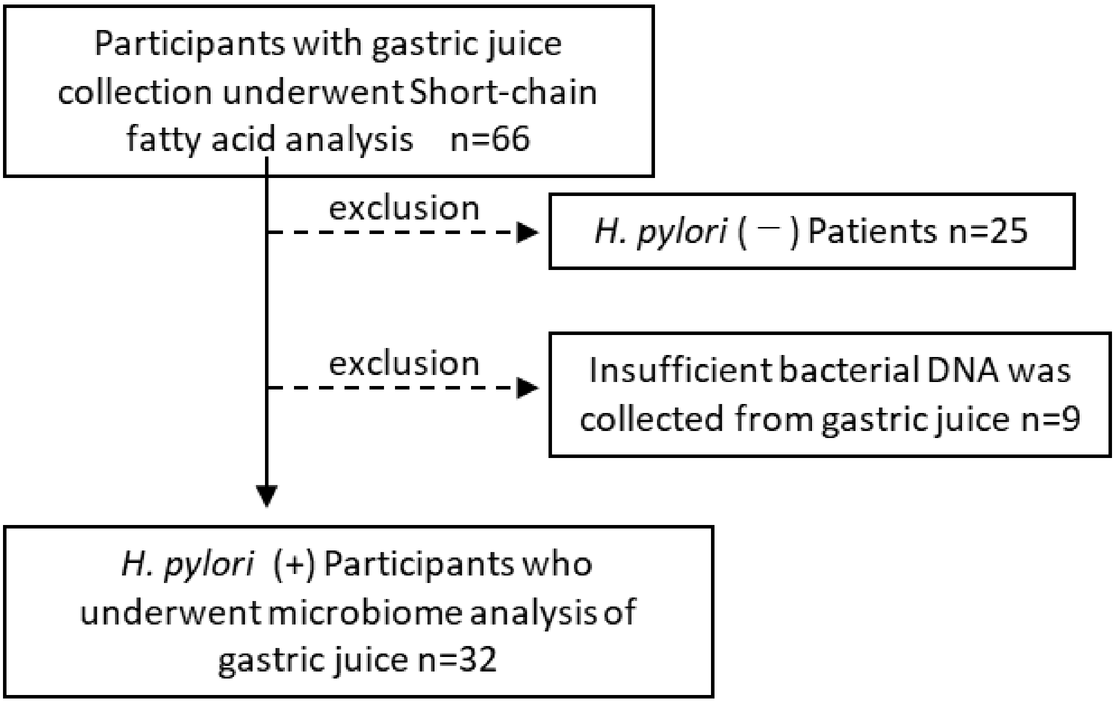

Supplementary Figure S1.

The inclusion criteria and study flow diagram. A total of 66 participants (44 male and 22 female) were enrolled. Of all participants, gastric juice microbiome analysis was performed on 32 individuals, excluding 25 uninfected with *H. pylori* and 9 individuals for whom insufficient bacterial DNA in the gastric juice could not be collected.

Supplementary Table S1.

Table S1. Basic patient characteristics by presence or absence of gastric cancer

	H. pylori + GC (+) (n=11)	*H. pylori* + GC (-) (n=30)	*P* value
Age (mean years± SD)	68.3±17.4	73.2±10.9	N.S. (.81)
BMI (mean years± SD)	20.2 ± 2.8	23.4 ± 2.8	< .01
Men n, (%)	8 (72.7)	22 (73.3)	N.S. (> .99)
Female n, (%)	3 (27.3)	8 (26.7)	
H. pylori current infection n, (%)	6 (54.5)	5 (16.7)	
H. pylori past infection n, (%)	5 (45.5)	25 (83.3)	
PPI/P-CAB n, (%)	5 (45.5)	11 (36.7)	N.S. (> .72)
